# Fostering Inflammatory Bowel Disease: Sphingolipid Strategies to Join Forces

**DOI:** 10.1155/2016/3827684

**Published:** 2016-01-05

**Authors:** Loubna Abdel Hadi, Clara Di Vito, Laura Riboni

**Affiliations:** Department of Medical Biotechnology and Translational Medicine, LITA-Segrate, University of Milan, 20090 Milan, Italy

## Abstract

Complex sphingolipids are essential structural components of intestinal membranes, providing protection and integrity to the intestinal mucosa and regulating intestinal absorption processes. The role of sphingolipid signaling has been established in numerous cellular events, including intestinal cell survival, growth, differentiation, and apoptosis. A significant body of knowledge demonstrates that intestinal sphingolipids play a crucial role, as such and through their signaling pathways, in immunity and inflammatory disorders. In this review, we report on and discuss the current knowledge on the metabolism, signaling, and functional implications of sphingolipids in inflammatory bowel disease (IBD), focusing on the different aspects of sphingolipid actions on inflammatory responses and on the potential of sphingolipid-targeted molecules as anti-IBD therapeutic agents.

## 1. Introduction

Complex sphingolipids, including sphingomyelin (SM) and glycosphingolipids (GSLs), are essential components of intestinal membranes, providing protection and integrity to the mucosa and regulating intestinal digestion and absorption processes. As in other organs, in the intestine, simple sphingolipids/sphingoids, which are intermediates of sphingolipid metabolism, are involved in the control of key cellular events such as survival, proliferation, differentiation, and apoptosis. Indeed, the metabolism of complex sphingolipid includes enzymes involved in different signaling pathways, which lead to the formation of bioactive molecules, including ceramide (Cer) and sphingosine (Sph), as well as their 1-phosphorylated derivatives ceramide-1-phosphate (C1P) and sphingosine-1-phosphate (S1P).

The role and impact of sphingolipids and sphingolipid-mediated signaling emerged in their relevance in intestinal disorders, when aberrations in their metabolism lead to an altered sphingolipid homeostasis. Herein, we review our current knowledge on the impact of sphingolipid disequilibrium on intestinal inflammation, focusing on inflammatory bowel disease (IBD).

## 2. Inflammatory Bowel Disease

The term IBD encompasses a group of common chronic inflammatory disorders affecting the gastrointestinal tract [1]. The major types of IBD are Crohn's disease (CD) and ulcerative colitis (UC). Despite some overlapping clinical features, these diseases are characterized by distinct inflammatory profiles, gut microbiota composition, and symptomatology [2, 3]. CD potentially affects any portion of the alimentary tract and is characterized by a discontinuous and ulcerous transmural inflammation, associated with complications (e.g., intestinal granulomas, obstructions, abscesses, strictures, and fistulas) [3]. In UC, a continuous inflammation involves only the superficial layers of the intestinal mucosa and is localized to regions of the gut most highly colonized by bacteria, specifically at the rectum and moving proximally along the large bowel [4].

**Figure 1:**
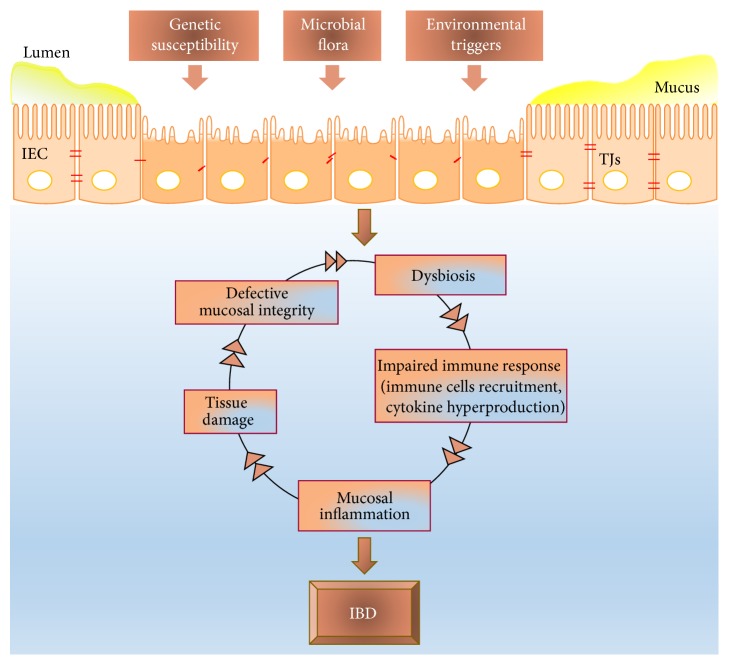
The pathogenesis of IBD. Genetic, microbial, and environmental factors participate to disrupt the intestinal barrier. The defective mucosal integrity starts a complex vicious cycle that leads to, enhances, and perpetuates IBD.

The pathogenesis of IBD is complex (Figure 1) and for many aspects remains unclear. The general hypothesis is that IBD develops as a result of a persistent alteration of intestinal homeostasis, leading to a perturbation of the balance between the intestinal mucosa and the gut microbiome [1]. Diverse factors, such as genetic, environmental, and immunologic variations, participate to and influence the onset and reactivation of this disease [4, 5]. There is compelling evidence that an inherited/acquired genetic predisposition that leads to barrier disruption and overreaction of the mucosal immune responses to enteric/environmental antigens are major factors contributing to the pathogenesis of IBD [6–8]. The dysregulated reaction of the mucosal immunity to normal intestinal microflora may be induced by defects in the epithelial barrier (increased intestinal permeability), adherence of bacteria, or expression of the “defensins” proteins.

The interaction among intestinal epithelial cells (IECs), intestinal microbes, and local immune cells plays a crucial role in the maintenance of the intestinal homeostasis and is disrupted in IBD, leading to overreaction of the mucosal immune response to normal intestinal microflora. Indeed, a common histopathological feature of IBD is an excessive immune activation, characterized by an exaggerated infiltration of mast cells, monocytes/macrophages, and polymorphonuclear leukocytes into the intestinal epithelium. This overabundance of immune cells is accompanied by continuous and dramatic production of proinflammatory stimuli, including cytokines, growth factors, and adhesion molecules, as well as of inflammatory mediators (especially those of the eicosanoid family) and reactive oxygen species (ROS) [9, 10]. All this results in the development of a severe and pervasive inflammation that promotes and exacerbates IBD.

## 3. Intestinal Sphingolipid Equilibrium

The small intestine is lined by a single layer of self-renewing IECs, which cover the surface of fingers-like projections called villi, and that of flask-like structures around the base of villi called crypts. The large intestine does not contain villi. Complex sphingolipids are present throughout the intestinal tract, with preferential localization in the apical membrane of polarized IECs, endowing its architecture with enhanced stability and digestive/absorptive capacity. Enterocytes of the small intestine are characterized by the selective abundance of SM and glucosylceramide (GlcCer), whose levels account for more than twofold that of the colonic mucosa and about 40% of total lipids [11]. The high content of sphingolipids in the small intestine is associated with selective enrichment and localization of several species in the apical membrane of the absorptive villous cells, which parallels the continuous process of mucosal cell differentiation throughout the crypt-villus axis [12]. Indeed, individual sphingolipids are differently distributed in villus and crypt cells, higher amounts of Sph, GlcCer, and GM3 being present in villi and Cer, trihexosyl-Cer, and GD3 ganglioside in crypts [13].

**Figure 2:**
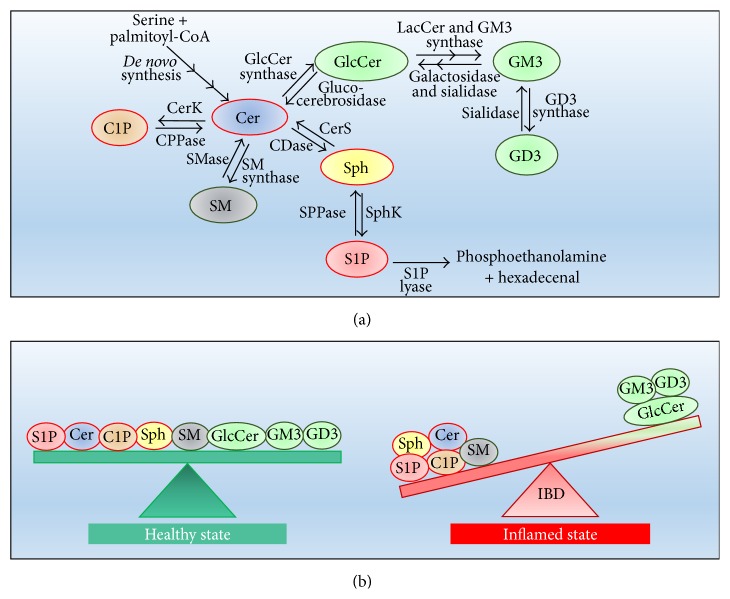
Metabolism and balance of intestinal sphingolipids in physiological conditions and IBD. (a) Interconnected pathways of intestinal sphingolipid metabolism. (b) In the healthy state, intestinal sphingolipids are in a “functional equilibrium” (left); in the inflamed state of IBD, an unbalance of this equilibrium occurs, favoring the inflammatory disease (right).

Sphingolipids have rapid turnover, and their levels are controlled by the balance between synthesis and degradation. As in most cells, the overall metabolism of intestinal sphingolipids is complex and intricate (Figure 2(a)) and involves multiple enzymes, also present in different isoforms and subcellular districts. Sphingolipids of the intestinal tract are synthesized via either “*de novo* pathway” (initiated by serine condensation with palmitoyl-CoA through serine palmitoyltransferase) or the “salvage pathway” (the recycling of free Sph derived from sphingolipid catabolism). In both pathways, the action of ceramide synthase (CerS) is required to produce Cer, and five of the six known isoforms of CerS are expressed in the intestinal mucosal cells [11]. The newly formed Cer in the endoplasmic reticulum is then transferred to the Golgi apparatus through either the protein (CERT) mediated transport (for SM biosynthesis) or a vesicle-mediated route (for both SM and GSLs formation) [14]. Two types of SM synthases (SMS) (SMS1 and SMS2) have been identified and cloned in the intestinal cells. SMS1 resides in the Golgi whereas SMS2 mainly presents at the plasma membrane. GlcCer synthase is highly expressed in the small and large intestine, rendering GlcCer the major intestinal GSL. GlcCer is also the substrate of galactosyltransferase and then of sialyltransferase abundant in villous cells, to provide lactosyl-Cer and GM3 [11].

Concerning sphingolipid catabolism, the intestine is characterized by the ability to hydrolyze both endogenous and exogenous (dietary-derived) sphingolipids. Intestinal SM degradation, catalyzed by sphingomyelinase (SMase) and ceramidase (CDase), has been the object of different studies. SMase and CDase can act both inside mucosal cells and as ectoenzymes, being present either in the intracellular environment or on the outer surface of the cell membrane or in the intestinal lumen. So far, three isoforms of SMase, acid (A-SMase), neutral (N-SMase), and alkaline (Alk-SMase), are figured out in the intestine with different compartmentalization. A-SMase (and in minor amounts N-SMase) is mainly localized in highly proliferating crypt cells, particularly of the proximal intestine, and appears to be mainly involved in the hydrolysis of SM internalized by endocytosis [11]. Alk-SMase is found preferentially in the middle part of the intestine, primarily at the brush border, and is recognized as the major enzyme for dietary SM digestion, even if it is also able to hydrolyze the plasma membrane SM of mucosal cells [15].

Besides SMase, also CDase is present in the intestine as three isoforms, including the neutral (N-CDase), the alkaline, and the acid one, the former exhibiting the highest catalytic activity in the presence of bile salts [16]. Although direct absorption of SM and Cer in humans cannot be excluded [17], there is ample evidence that free Sph, produced through Alk-SMase and N-CDase digestion, is the major absorbed product of dietary SM. Once internalized into the enterocytes, Sph is rapidly metabolized, mainly to S1P by sphingosine kinase (SphK). In the small intestine of mice, the total SphK activity was shown to be about twofold that of colon, and SphK1 contributed approximately 40% to the total kinase activity in small intestine, whereas it was the prevalent isoform in colon [18]. Interestingly, an unidentified SphK with high activity toward phytosphingosine was reported to be the prominent SphK in the small intestine [18]. S1P lyase that degrades S1P to phosphoethanolamine and palmitaldehyde is expressed at high levels in the intestinal mucosa [19], most probably to reduce the level of S1P (and Sph) derived from the digestion of dietary sphingolipids.

This intricate network of metabolic reactions leads to the physiological presence of multiple sphingolipid molecules, which play fundamental roles in the digestive, absorptive, protective, and defense properties of the intestine.

## 4. Aberrant Sphingolipid Metabolism Leads to a “Proinflammatory” Sphingolipid Pattern in IBD

There is compelling evidence that dysregulated production of several sphingolipid molecules occurs along with IBD and contributes as a major factor to the pathogenesis and maintenance of this disorder. Indeed, sphingolipid metabolism and the cellular level/distribution of different sphingolipids exhibit significant changes in IBD. These variations not only alter the healthy sphingolipid equilibrium essential for intestinal functions, but also implicate cell-signaling responses that precipitate the pathology.

Accumulating pieces of evidence demonstrate that a significant increase in some sphingolipid species, including SM, Cer, and the sphingoids mediators C1P and S1P, is associated with IBD and is counterbalanced by the decrease of other molecules, especially GlcCer and GM3 (Figure 2(b)). Indeed, SM and Cer are significantly increased in a colitis mouse model [20], as well as in feces of animals with dextran sulfate sodium- (DSS-) induced colitis (an experimental model of IBD) [21]. In agreement, and of relevance, high levels of SM and Cer were reported in the ileum from CD patients [22], providing evidence that SM and Cer generation accompanies and possibly aggravates chronic intestinal inflammation. Different enzymatic alterations appear to contribute to the increased SM in IBD. First, a decrease in Alk-SMase activity was demonstrated in human chronic colitis [23], possibly acting as a key mechanism to induce SM accumulation. In addition, colon samples from mice with a DSS-induced IBD exhibit downregulation of N-SMase, and, of relevance, concomitant upregulation of SMS2 [20].

Both the increase of glucocerebrosidase (responsible for GlcCer hydrolysis to Cer) and CerS activation were found in IBD and were suggested to be responsible for Cer elevation [24]. Moreover, in colon cancer cells, tumor necrosis factor *α* (TNF-*α*), a proinflammatory cytokine that plays a pivotal role in IBD, was shown to upregulate the* de novo* pathway of sphingolipid biosynthesis [25], and it is conceivable that this effect could contribute to Cer and SM accumulation in IBD. A further study on mice revealed that also Sph level is elevated in colon inflammation [26], most likely as a consequence of the increased expression and activity of N-CDase. Indeed, it was shown that N-CDase expression and activity increase in colon epithelium of mice during DSS-induced IBD [26]. On the contrary, it was shown that the ganglioside content of inflamed intestinal mucosa is significantly decreased [27]. Whether enhanced catabolism of gangliosides and/or decreased biosynthesis from Cer are responsible for ganglioside depletion in IBD remains unknown. In a rat model of intestinal inflammation, the 1-phosphorylated forms of Cer (C1P) and Sph (S1P) were shown to increase in a time-dependent fashion with inflammation [28]. The increase of S1P levels is of particular relevance in IBD colon and occurs not only in the intestine but also in blood, as a consequence of the enhanced expression of SphK1 [29]. On the contrary, the deficiency of SphK2 was shown to significantly reduce IBD severity [30].

It is unknown whether alterations of sphingolipid metabolism precede the onset of the chronic inflamed state of IBD, or the inflammatory condition induces them. It appears plausible that both conditions are operative, sphingolipid aberrations favoring and on their turn being favored by the IBD condition.

## 5. Sphingolipid Unbalance: A Multiarmed Force of IBD

**Figure 3:**
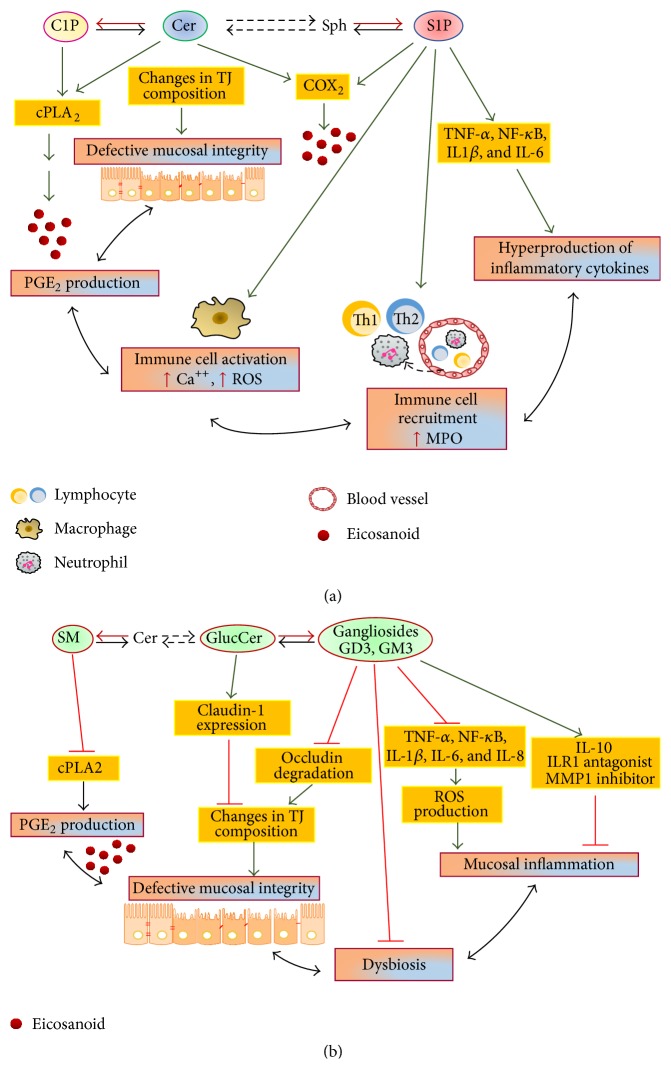
Inflammatory roles of sphingolipids in IBD. (a) Simple sphingolipids (C1P, Cer, and S1P) act as potent proinflammatory messengers, favoring and exacerbating the IBD condition. (b) Complex sphingolipids (SM and GSLs) exert several anti-inflammatory effects.

IBD has many tiers of initiation, progression, and evolvement to reach the summit of intestinal damage. The multiple and significant alterations of sphingolipids and bioactive sphingoids level associated with IBD result in a variety of effects on these tightly interrelated tiers, including the epithelial barrier integrity, immune cell targeting and signaling, and innate/adaptive immune responses (Figure 3).

### 5.1. Sphingolipid Alterations Favor the Disruption of the Intestinal Barrier in IBD

A critical function of the intestinal epithelium is to form a selective permeable barrier that allows the appropriate absorption of nutrients but limits the permeation of noxious agents, such as pathogens, toxins, and antigens, from the luminal environment [31]. This specialized permeable barrier is achieved by different structures, including mucus and intercellular tight junctions (TJs), which control luminal molecules-intestinal cells interactions and paracellular permeability [32]. It is recognized that the disruption of the intestinal TJ barrier, followed by the permeation of luminal noxious agents, and perturbation of the mucosal immune system and inflammation, acts as a key trigger of IBD development [33]. IBD patients exhibit increased intestinal permeability, which favors bacterial/viral infections and thus onset/relapse of IBD. Observations on IBD patients pointed out the role of bacteria in this inflammation, and antibiotics were found to be effective in some patients with IBD [34]. In agreement, most mouse models of colitis require intestinal bacteria to occur [35].

It is noteworthy that the intestinal permeability is also influenced by the content and pattern of IEC sphingolipids (Figure 3). Both Sph and its N-acylated derivative Cer are elevated in IBD and emerged as antibarrier effectors (Figure 3(a)). By using fumonisin B1 (a fungal inhibitor of CerS that leads to Sph accumulation and depletion of complex sphingolipids), it was found that the toxin induces a primary defect in barrier function, increasing intestinal epithelial permeability [36]. In addition, fumonisin B1 treatment in pigs resulted in a significant increase of intestinal colonization by pathogenic* E. coli* [37]. As far as Cer is concerned, it is recognized that it selectively accumulates in cholesterol- and sphingolipid-enriched membrane microdomains, which also include TJs [33]. The packing of Cer in these microdomains alters the lipid rafts composition, contributing to a disturbed barrier function [38]. In agreement, the neutralization of cell surface Cer prevented the loss of barrier function induced by platelet-activating factor (PAF) [38].

Opposite to the negative effects of Sph and Cer at the intestinal barrier, GSLs (gangliosides and GlcCer), enriched on the luminal membrane of enterocytes, protect intestinal mucosa from injuries induced by bile salts, gastric juice acidity, and toxins/pathogens entry (Figure 3(b)). In particular, through their negative charge, gangliosides such as GM1 and GM3 are able to bind, and then inactivate, the toxins of* Vibrio cholerae*,* E. coli*, and* Shigella* [39–41]. Of interest, these antibacterial effects can be improved by dietary gangliosides. A recent* in vivo* study on lipopolysaccharide- (LPS-) induced inflammation revealed that dietary gangliosides inhibit the degradation of gut occludin, a major protein of intestinal TJs [42], implicating them in the intestinal barrier properties. In addition, dietary GlcCer was shown to induce the expression of claudin-1, thus improving TJ properties [43].

Overall, it emerges that the significant reduction of GSLs, especially GM3 and GlcCer, associated with the concomitant elevation of Sph and Cer, markedly concurs to alter the permeability properties of the intestinal epithelium in IBD.

### 5.2. Sphingolipids and Cytokine Networks in IBD

A well-recognized feature of IBD is the overproduction of proinflammatory cytokines, mainly represented by TNF-*α*, interleukin- (IL-) 6, and IFN-*γ* [44]. In the IBD intestine, excess of macrophages and subpopulations of macrophages not normally present in the lamina propria of the intestine is present, indicating ongoing recruitment to the inflamed bowel. The abundance and activation of these innate and adaptive immune cells in the intestinal mucosa result in increased local levels of proinflammatory mediators [44]. In active IBD, an elevated expression of TNF-*α* occurs, consequent to the high density of TNF-*α* producing cells, especially macrophages within the lamina propria [45]. The high level of TNF-*α* contributes to the inflammatory tissue damage, by impairing the integrity of epithelial and endothelial membranes and increasing the recruitment of inflammatory cells [46].

Different studies implicate a strong network connection between cytokines and different sphingolipids as critical in the regulation of inflammatory pathways and immune reactions in IBD. In this connection, SphK1 emerged as key actor, being activated by several cytokines, among which TNF-*α* plays a prominent role [47]. SphK1 acts as important mediator of various cellular processes in IBD, most likely downstream of TNF-*α* and upstream of cyclooxygenase- (COX-) 2. Indeed, in a DSS-induced colitis model, Snider et al. [29] reported that SphK1 depletion failed to induce colonic COX-2 and the consequent prostaglandin (PG)E2 production, which contributes to the exacerbation of the inflammatory colon damage. In the same study, it was reported that S1P produced by SphK1 is able to promote neutrophil infiltration into the crypts and lamina propria of the colon [29]. Thus, S1P emerged as a chemoattractant for granulocytes in the inflamed intestine either directly or through the production of other local chemoattractants, and in both cases the SphK1/S1P pathway promoted the tissue inflammatory reaction. In a follow-up study, the same group provided further insights into the distinct roles of hematopoietic (bone marrow-derived) and extrahematopoietic (intestinal) SphK1/S1P pathway in the pathogenesis of IBD [48]. Hematopoietic-derived SphK1 was found as major contributor to circulating S1P, which, on its turn, was implicated in lymphocyte egress from the spleen, and circulating neutrophil increase [48]. This led to a high neutrophil/lymphocyte ratio, a steady indicator of systemic inflammation in colitis [49]. In addition, both hematopoietic and extrahematopoietic S1P were involved in the increased expression of IL-1*β* and IL-6, as well as in the phosphorylation of STAT3, a tightly regulated transcription factor implicated in chronic colitis associated cancer [48, 50]. As far as the extrahematopoietic SphK1/S1P is concerned, this was shown to be essential for the autocrine induction of COX-2 in the colon tissue [48].

Although the absence of SphK1 was protective against DSS-induced colitis [51], SphK2 depletion resulted in enhanced proliferation and proinflammatory cytokine production, and thus IBD progression [30, 51]. Indeed, knockout mice for SphK2 were shown to develop more severely damaged and necrotic colonic mucosa, with pronounced loss of crypt structures and extensive infiltration of inflammatory cells, eosinophils, and neutrophils [30]. In SphK2(−/−) mice, mesenteric lymph node cells overproduced proautoimmunity cytokines, and T-cells induced more rapid and robust IBD in scid recipients which enhanced the pathological phenotypes of colitis severity, suggesting that SphK2 negatively modulates inflammatory responses and significantly reduces IBD severity [30]. These findings support the hypothesis that therapeutic enhancement of SphK2 activity and/or depletion of SphK1 may be effective treatments for the exuberating inflammation of IBD.

Different reports demonstrate that Cer generated by SMase is able to exert powerful effects, even opposite, on IBD cytokine production and signaling. In the inflammatory processes of the gastrointestinal tract, the infiltration of inflammatory cells into the intestinal mucosa leads to a marked increase of IL-1*β* and then to NF-*κ*B activation [44]. Both IL-1*β* and NF-*κ*B exert detrimental effects, by inducing the expression of proinflammatory mediators that orchestrate and sustain the inflammatory response, finally determining tissue damage. A study by Homaidan et al. [52] on murine IECs demonstrated that IL-1*β* activates N-SMase through NF-*κ*B, thus inducing significant accumulation of Cer. Cer was found to mimic IL-1*β* in enhancing PGE2 generation through COX-2 induction and by promoting the rise of chloride and water secretion, a typical alteration of IBD patients [52, 53]. Cer generated by N-SMase was shown to induce apoptosis in human colon HT-29 cells, through NF-*κ*B activation and IL-8 expression [54]. In addition, also the A-SMase-derived Cer was apoptotic, but by activating NF-*κ*B on distinct *κ*B complexes [54]. Besides IL-1*β* and TNF-*α*, also LPS induces the release of inflammatory cytokines from monocyte/macrophages via A-SMase activation, and Cer generation leads to the expression of a variety of genes and results in inflammatory responses [55].

It is recognized that Cer can exert opposite effects depending on its enzymatic origin and/or site of production [56]. Indeed, opposite to the proinflammatory effects of Cer produced by N- and A-SMase in the intestine, Cer generated by Alk-SMase was shown to promote intestinal anti-inflammatory pathways. In fact, the conversion of the apical membrane SM to Cer by intestinal Alk-SMase was found to inhibit cholera toxin endocytosis and host cells intoxication [57]. Further support for the anti-inflammatory action of intestinal Alk-SMase was provided by a report showing that, in rats with DSS-induced colitis, the intrarectal instillation of Alk-SMase induced TNF-*α* inhibition, significantly reduced the inflammation score, and protected the colonic epithelium from inflammatory destruction [58]. These studies, together with the finding that Alk-SMase is downregulated in IBD [23], support the notion that the decrease of this anti-inflammatory enzyme may be of relevance in IBD pathogenesis and progression.

The excessive cytokine production in IBD appears to be favored also by the decrease of gangliosides occurring in this disease. It is recognized that gangliosides in the apical intestinal surface can influence numerous processes, including microbial attachment, toxin production, and infectivity of several intestinal pathogens (Figure 3(b)), and increased ganglioside catabolism occurs in IBD and results in increased proinflammatory signaling [59]. Intriguingly, dietary gangliosides emerged as effective elements in reducing proinflammatory mediators of IBD. Indeed, an induced ganglioside increase was shown to inhibit both TNF-*α* and IL-1*β* proinflammatory signals in rats [27]. Moreover, in a cultured infant bowel exposed to LPS in hypoxic conditions, gangliosides were able to reduce both IL-6 and IL-8 production [60].

### 5.3. Cross-Communication of Sphingolipid Messengers with Eicosanoid and Glycerolipid Signaling

Aberrant production of bioactive lipids of the eicosanoid family, especially PGs, was identified to drive chronic inflammation by dysregulating signaling pathways and/or cellular events and leading to abnormal immune functions [61]. Eicosanoids, including PGs, are generated from arachidonic acid, released from cell membrane phospholipids mainly by phospholipase-A2 (PLA2). Arachidonic acid is then converted to PGs via the COX pathway. Of the two known COX isoforms (COX-1 and COX-2), it is COX-2 that is induced during acute or chronic inflammation [62].

Increased PG production occurs within the gastrointestinal mucosa of patients with IBD, and in experimental models of IBD [63]. As a consequence, PGs and especially PGE2 act as proinflammatory factors and tool in IBD. Different studies progressively revealed a key role of sphingolipids in the modulation of eicosanoid production in inflammatory processes [64] (Figure 3(a)). Initial studies showed that Cer and Sph were able to induce PGE2 production [65]. Cer was shown to enhance cytosolic PLA2 (cPLA2) activity and PG formation through direct binding to the enzyme [66]. Subsequent studies demonstrated that both C1P and S1P are involved in the regulation of PGE2 production [67–69], by acting through different mechanisms. C1P was shown to activate cPLA2 either directly or by promoting its translocation to intracellular membranes [70–72]. After binding to different subtypes of its receptors, S1P acts downstream of Cer, by causing strong and prolonged expression of COX-2 [73], through inducing transcription and mRNA stabilization [74, 75].

The proinflammatory cytokines IL-1*β* and TNF-*α* were shown to regulate both cPLA2*α* and COX-2 to promote SM metabolism and to produce C1P and S1P formation [76]. This signaling pathway starts with A-SMase activation, which results in the reduction of the SM-mediated inhibition of cPLA2*α*. Moreover, the two cytokines activate SphK1 and ceramide kinase 1, the Cer pool generated by A-SMase finally promoting both C1P and S1P biosynthesis. In response to proinflammatory cytokines, C1P and S1P exhibit a significant synergistic effect on the activation of cPLA2*α* and COX-2, and thus on the production of the key inflammatory mediators PGE2 [67].

Further support to IBD maintenance derives from the different catalytic activities exhibited by the Alk-SMase. This enzyme is able to hydrolyze not only SM, but also other choline phospholipids, including lysophosphatidylcholine, with reduction of lysophosphatidic acid, and PAF [15, 77]. Thus, the Alk-SMase reduction of IBD appears to be functional to signaling alterations that potentiate IBD, by reducing the anti-inflammatory actions exerted by SM and PAF and by promoting the proinflammatory lysophosphatidic acid.

Although much remains to be understood about these multiple signaling interactions, the actual knowledge linking sphingolipids with eicosanoids and glycerolipid messengers provides a new conceptual view on how alterations of sphingolipid signaling in IBD act not only as such but also through regulation of other signaling molecules. This leads to increased efficacy in promoting and maintaining the chronic state of IBD.

## 6. Sphingolipids: Potential Therapeutic Benefits/Targets in IBD

The global incidence of IBD is increasing with time [78]. Actually, a step-up strategy is used in the management of IBD, therapeutic treatments including 5-aminosalicylic acid, glucocorticoids, immunomodulators, and anti-TNF agents [79]. Despite advancements in therapy with the introduction of anti-TNF agents [80, 81], IBD gradually causes serious effects in terms of morbidity, work disability, and quality of life [82]. The finding that multiple sphingolipid alterations are associated with IBD has prompted investigations on the targeting of sphingolipids as a potential field of therapy for managing progression and morbidity of this disease.

### 6.1. Dietary Bioactive Sphingolipids: A Gift from Nourishment

It is generally accepted that dietary components are involved in immune regulation. The intestinal immune system, especially, seems to be directly affected by the digestion and absorption of dietary compounds and, among them, dietary lipids have emerged as major determinants of the intestinal immune responses, mainly after conversion into lipid mediators [83]. Indeed, as described above, sphingolipid metabolites act as multifaceted bioactive molecules in IBD, and different studies demonstrated that dietary complex sphingolipids, including gangliosides and SM, possess the potential of effective compounds.

Initial studies on dietary gangliosides demonstrated that the sialylated GSLs of human milk exert beneficial effects in infant bowel infections, through enterotoxin-inhibiting activity and maintenance of the intestinal barrier integrity [84]. Thereafter, it was shown that milk gangliosides were able to inhibit adhesion and suppress the growth of* E. coli*. In particular, an infant formula, specifically enriched with GD3 and GM1 gangliosides, lowered* E. coli* content and concomitantly increased that of bifidobacteria, suggesting a ganglioside role in the development of intestinal immunity [85]. Furthermore, Schnabl et al. [60] demonstrated that ganglioside preexposure to LPS treatment reduces bowel necrosis and endothelin-1 production in response to LPS, by suppressing infant bowel production of different proinflammatory mediators, such as nitric oxide, leukotriene B4, PGE2, hydrogen peroxide, IL-1*β*, IL-6, and IL-8. Moreover, very recently, it was reported that gangliosides, especially GD3, ameliorate intestinal injury not only by suppressing proinflammatory mediators, but also by upregulating anti-inflammatory molecules, such as chemokines and the cytokine IL-10 [86] (Figure 3(b)).

Besides gangliosides, also dietary SM was shown to exert biomodulatory activities in intestinal inflammation, though with contrasting results. In the mouse colon, it was observed that the simultaneous administration of SM and DSS prevents the increase of myeloperoxidase (MPO) activity, a marker of neutrophil influx into the inflamed colon [87]. Moreover, Mazzei et al. [88] recently reported that dietary SM decreased colonic inflammatory lesions and disease progression in mice with DSS-induced colitis. On the contrary, two recent papers by Hausmann's group showed that, after metabolism to Cer, dietary SM triggers apoptosis in murine IECs and aggravates intestinal inflammation in acute DSS-induced colitis and in IL-10 knockout mice [21, 89]. The reasons of these opposite findings on dietary SM in intestinal inflammation remain to be clarified. Although it cannot be excluded that the use of different mouse strains may be responsible for the anti- and proinflammatory effects of dietary SM, it appears likely that the different composition and intestinal hydrolysis of the administered SM (of milk and egg origin, resp.) play a major role.

Taken together, these findings suggest that the dietary assumption of specific types of complex sphingolipids might be beneficial as preventive/therapeutic strategy against IBD.

### 6.2. Regulation of Sphingolipid Metabolites by Dietary Materials

In addition to dietary sphingolipids, the assumption of dietary compounds able to modify the intestinal sphingolipid metabolism has emerged as an intriguing possibility to influence the IBD status.

It has been demonstrated that a diet enriched with fibers and fats is able to influence the expression of both intestinal SMases and CDases. Psyllium, a water soluble fiber partly digested by bacterial flora, increased the activity of Alk-SMase and decreased that of A-SMase and N-CDase alleviating inflammation, whereas a high-fat diet exerted opposite effects [90]. Moreover, in IL-10 KO mice with colitis, it was demonstrated that the VSL#3 probiotic upregulates Alk-SMase activity, suggesting its potential as anti-inflammatory agent [91].

Luteolin, a tetrahydroxyflavone present in a variety of vegetables, fruits, and medicinal herbs, has been shown to function as an antioxidant, anti-inflammatory, and anticancer agent [92]. Very recently, we reported that dietary luteolin is able to unbalance the sphingolipid rheostat by inhibiting both S1P biosynthesis and Cer traffic in colon cancer cells [93], suggesting its dietary introduction/supplementation as a potential strategy to improve inflammation in colorectal cancer. These findings prompt further investigations on dietary molecules able to modulate sphingolipid metabolism as anti-IBD foods.

### 6.3. Targeting Sphingolipids: A Potential Anti-IBD Strategy

The discovery of alterations of sphingolipid metabolism and signaling in IBD paved the way for the discovery of pharmacological approaches to prevent/treat this disease. Up to now, several compounds have been discovered to manipulate the SM cycle and S1P metabolism/receptors, and these were evaluated for their potential therapeutic benefit generally in inflammation, and especially in IBD.

So far, the most high-profile drug that improves inflammation via S1P receptor (S1PR) modulation and is assessed* in vivo* is the fungal metabolite fingolimod (FTY720). Differently from typical immunosuppressive drugs, its mode of action is not via inhibition of T-cell function, but via downregulation of S1P1 on lymphocytes, leading to the inhibition of their egress from the lymph nodes [94]. As a consequence, FTY720 reduces the number of T-cells circulating between lymph nodes and the peripheral site of tissue inflammation. In agreement with this action, Mizushima et al. [95] demonstrated the efficacy of FTY720 in decreasing the severity of spontaneous colitis in the IL-10-deficient mouse model. FTY720 induced accelerated homing and sequestration of T-cells into Peyer's patches and mesenteric lymph nodes, resulting in a reduction of CD4+ T lymphocytes into the lamina propria [95]. An additional study provided new evidence on the immunosuppressive properties of FTY720. In particular, in a chemically induced mouse model of colitis, FTY720 treatment markedly reduced the severity of intestinal inflammation [96]. This therapeutic effect of FTY720 was associated with downregulation of different proinflammatory cytokines, paralleled by a functional activity of CD4+CD25+ regulatory T-cells and prominent upregulation of anti-inflammatory mediators [96].

The finding that S1P1 modulation was the major mechanism of FTY720's efficacy prompted the search for second-generation compounds, with high selectivity to S1P1, and lower clinical adverse events (linked to its agonistic activity to S1P3), such as symptomatic bradycardia [97]. Among new drugs, KRP-203, S1P1/4/5 agonist prodrug with a molecular structure resembling FTY720 with high selectivity to S1P1, was developed for immunomodulation in autoimmune diseases and organ transplantation [98]. In the IL-10-deficient mouse, a validated model of human IBD, KRP-203 regulated not only T-cell response but also B-cell one [99]. Its treatment resulted in a significant reduction in the severity of colitis and in the number of CD4(+) T-cell and B220(+) B-cell subpopulations and T-helper 1 (Th1) cytokine production in colonic mucosa, a histopathological hallmark of IBD [99]. Thus, the use of KRP-203 emerged as an option to be tapped into effective immunointervention in IBD.

W-061, a novel and potent S1P1 agonist, structurally different from Sph and active* in vivo* without undergoing phosphorylation, showed effectiveness for alleviating multiple aspects of chronic intestinal inflammation in DSS-induced colitis, such as preventing mucosal thickness and mucin depletion induced by DSS. The novel effect attributed exclusively to W-0671 as S1PR agonist is the suppression of Th17 and Th1 increase in the lamina propria, by the sequestration of these cells into secondary lymphoid tissues [100]. It has been recently demonstrated that SEW 2871, another selective S1P1 agonist, ameliorates colitis in IL-10-deficient mice, by reducing proinflammatory cytokines production and promoting the expression of TJ typical proteins [101]. A further, highly specific S1P1 agonist, named RPC 1063, has been successfully tested in animal models of IBD and is currently being used as an oral therapeutic in phase II clinical trials of UC [102].

Besides S1PR antagonists, other drugs exhibiting a promising therapeutic benefit in IBD are those targeting the S1P axis and acting as SphK inhibitors. Different studies demonstrated that the orally available inhibitors of SphKs, ABC747080 and ABC294640, are effective in inhibiting the severity of colitis [29, 103, 104]. This protection is likely due to the significant decrease, induced by the inhibitors, of a broad spectrum of inflammatory mediators, such as TNF-*α*, COX-2, IL-1*β*, IFN-*γ*, MPO, and IL-6, including S1P. Moreover, SphK inhibitors were able to attenuate the effects of TNF-*α*-induced increase in the expression of the adhesion proteins physiologically involved in leukocyte recruitment.

Of notice, in both acute and chronic models of UC, it has been reported that SphK inhibitors and Dipentum, an FDA-approved anticolitis drug, exhibit similar effects in reducing inflammation extent and severity, with the best results in a combinatory delivery [103]. These results are encouraging and shed light on a possible combination of SphK inhibitors and anti-IBD drugs for further clinical IBD improvement. Overall, it emerged that SphK is an excellent target for the development of new anti-IBD drugs.

Additional studies investigated the SM cycle as therapeutic target in IBD. By using SMA-7, an inhibitor of A-SMase and N-SMase, it was found that, besides suppressing Cer production, it was effective in inhibiting NF-*κ*B activation and inflammatory cytokine release from macrophages [55]. In murine models of colitis, the oral administration of SMA-7 induced a significant decrease in the NF-*κ*B-dependent cytokine levels and reduced alterations and damage in the colonic mucosal layer [55]. Whether N- and A-SMases play a different role in the regulation the IBD inflammation and the contribution of Cer inhibition and/or S1P reduction as key effectors of the SMA-7 effect remain to be clarified.

Finally, also glycosylated sphingolipids recently emerged as compounds of potential benefit in IBD. In particular, cerebroside D, a glycoceramide compound from fungal culture, was presented for its immunosuppressive activity and efficiency in improving DSS-induced colitis in mice [105]. Its anti-inflammatory mechanism involves multiple effects against activated T-cells in colon, such as regulation of the cytokine profile and apoptosis induction in activated T-cell effectors. Thus, by interfering with naïve T-cell activation, cerebroside D appears to be of relevance for IBD management.

## 7. Closing Remarks

Taken together, a huge and increasing volume of experimental evidence points to the importance of sphingolipids/sphingoids molecules, and their enzymes, in IBD development and fate. However, at present, several gaps in our knowledge and many questions need to be understood better. Among them, more knowledge is required on the mechanisms underlying the* in vivo* digestion/absorption of sphingolipids in the gastrointestinal system, as well as on the effective concentrations shifting them from food components to dietary bioactive nutrients. The influence of specific lipids, and their modifying enzymes, on intestinal lipid-barrier control is an area of great promise that needs to be investigated further to develop novel treatment strategies that strengthen the intestinal barrier and possibly halt, or at least slow down, the initial alterations leading to IBD. It will be also of relevance to better understand the multifaceted role of sphingolipid molecules in IBD and their subcellular localization, as well as cross talk with other intestinal factors involved in this disease. The potential of diet components in changing the sphingolipid amount and pattern in the gut remains an open question. In the “future food” field, it is a hard challenge to find out new compounds targeting sphingolipid metabolism and able to self-actualize, or to combine with other drugs, for effective IBD treatment with minimal side effects. Based on the preclinical findings of the anti-IBD potential of sphingolipid, hard work remains to establish their efficacy and to upgrade and improve treatments to clinical endpoints. Deeper understanding of the proper role of sphingolipids and their enzymes in controlling the intestinal properties and in promoting the pathogenesis and progression of IBD will generate new perspectives in the development of “sphingolipid-centered” therapeutic strategies that control the onset and perpetuation of this disabling inflammation.

## Abbreviations

IBD: Inflammatory bowel disease
SM: Sphingomyelin
GSLs: Glycosphingolipids
Cer: Ceramide
Sph: Sphingosine
C1P: Ceramide-1-phosphate
S1P: Sphingosine-1-phosphate
CD: Crohn's disease
UC: Ulcerative colitis
IECs: Intestinal epithelial cells
ROS: Reactive oxygen species
GlcCer: Glucosylceramide
CerS: Ceramide synthase
SMS: SM synthases
SMase: Sphingomyelinase
CDase: Ceramidase
Alk-SMase: Alkaline sphingomyelinase
N-SMase: Neutral sphingomyelinase
A-SMase: Acid sphingomyelinase
N-CDase: Neutral ceramidase
SphK: Sphingosine kinase
DSS: Dextran sulfate sodium
TNF-*α*: Tumor necrosis factor alpha
IFN-*γ*: Interferon-*γ*
TJ: Tight junction
LPS: Lipopolysaccharide
PAF: Platelet-activating factor
IL: Interleukin
COX: Cyclooxygenase
NF-*κ*B: Nuclear factor kappa B
PG: Prostaglandin
cPLA2: Cytosolic phospholipase-A2*α*
S1PR: S1P receptor.
